# Intelligent Hotel Guidance System via Face Recognition Technology

**DOI:** 10.3390/s23229078

**Published:** 2023-11-09

**Authors:** Chenlu Bao, Yongjie Yang, Zhiliang Wang, Peng Xu

**Affiliations:** 1 School of Information Science and Technology, Nantong University, Nantong 226019, China; 2110310008@stmail.ntu.edu.cn (C.B.); yang.yj@ntu.edu.cn (Y.Y.); wangzl@ntu.edu.cn (Z.W.); 2 Nantong Research Institute for Advanced Communication Technologies Co., Ltd., Nantong 226019, China

**Keywords:** hotel management, intelligent systems, internet of things, face recognition, MQTT

## Abstract

In modern large hotels, due to a large number of rooms and complex layouts, it is difficult for customers to find rooms, which increases a lot of workloads for hotel attendants to guide. In this paper, a hotel intelligent guidance system based on face recognition is designed. After entering the customer’s facial photos, the room guidance and customer management are carried out through face recognition. With this, hotels can move toward card-free management, green environmental protection, and save on resources. With these improvements, hotel management will be card-free and green. Each monitoring device of the system adopts dual STM32 core architecture, in which STM32H7 is responsible for face recognition, while STM32L4 is the main control chip, which is responsible for data exchange, guest room guidance and other work. The monitoring master not only guides, but also uploads customer check-in information to the cloud platform to facilitate the management of the hotel. The system adopts contactless information collection and guidance, which improves the intelligence and humanization of the hotel, and has a good application prospect.

## 1. Introduction

The Internet of Things (IoT) greatly promoted the progress of Industry 4.0, and has spread to home automation, health care and other industries [1,2,3]. With the continuous progress of hardware technology, information technology and data technology, hotel management technology is also constantly innovating, and hotel management tends to be digital and intelligent [4]. The use of intelligent technology injects new vitality into the hotel, not only providing customers with high-quality service, but also making the hotel operate more efficiently and save costs [5,6]. As a part of the economic strength of the city, the growth of the number of intelligent hotels can also promote the development of tourism and economy [7]. From the customer’s point of view, fewer opportunities for direct contact can reduce the spread of novel coronavirus, thus effectively ensuring the safety of life. Therefore, the emergence of intelligent hotels is the inevitable trend of the development of science and technology, and an irresistible wave of innovation.

Elkhwesky [8] described in detail the great opportunities and challenges that IoT technology brings to the hotel industry. Many existing hotel intelligent design schemes refer to the smart home scheme or smart city scheme to realize the intelligent facilities in the room. Xu [9] focuses on software system design, which is based on big data for customer information analysis. The system aims to connect multiple independently controlled rooms to improve the efficiency of the system [10]. The system remotely monitors the electricity consumption of each room through the wireless network [11]. The design uses the database to manage customer data and hotel information, which is convenient for customers to book rooms [12]. The system uses the design concept of smart city to intelligently upgrade the hotel [13]. IoT could assist the staff in kitchens to maximize their efficiency and know electricity and water consumption [14]. Customers can control the electronic devices in the room through voice, text and tablet computers [15,16]. One of the major challenges of applying IoT in hotels is the cost [17,18].

With the development and maturity of face recognition (FR) technology [19,20] and artificial intelligence, non-contact systems based on face recognition are favored. Classical face recognition algorithms include face recognition algorithms based on geometric feature extraction (GFE), principal component analysis (PCA), convolutional neural networks (CNN), and so on [21]. GFE describes the relationship between basic shape and structure, ignores local fine features, and is suitable for facial expression recognition. Face recognition based on PCA or CNN puts forward high requirements for the computing power of hardware devices. In the current face recognition systems, the front-end camera is only responsible for image acquisition, and face images are all sent to the central computer and processed there. Thus, there will be problems such as network congestion. All in all, our contributions are as follows:

- We present a novel train of thought to provide intelligent service. According to the actual situation and needs of the hotel industry, we study the composition and architecture of the hotel intelligent system, and analyze the need for hotels to provide face recognition guidance in a complex environment. Combined with the advantage of edge computing, face recognition modules are deployed at the edge of the system network, which greatly improves the processing efficiency and reduces the load on the central server.
- We propose an intelligent guidance system based on FR. The system combines the IoT technology, cloud technology and the Message Queue Telemetry Transport (MQTT) communication protocol to achieve the exchange of information between customers and the hotel management system, including real-time storage, rapid identification, contactless guidance, location monitoring, historical data query, and so on.
- We design a self-designed software and hardware system, build a complete customer to management system, and realize non-contact intelligent guidance. The face recognition part uses open-set evaluation, which is more referential. This system can be connected with the identity information database of public security organs. Combined with respiratory monitoring, this system facilitates the trajectory tracking of the current COVID-19 epidemic risk personnel.

## 2. Intelligent Hotel Guidance System Design

**Figure 1:**
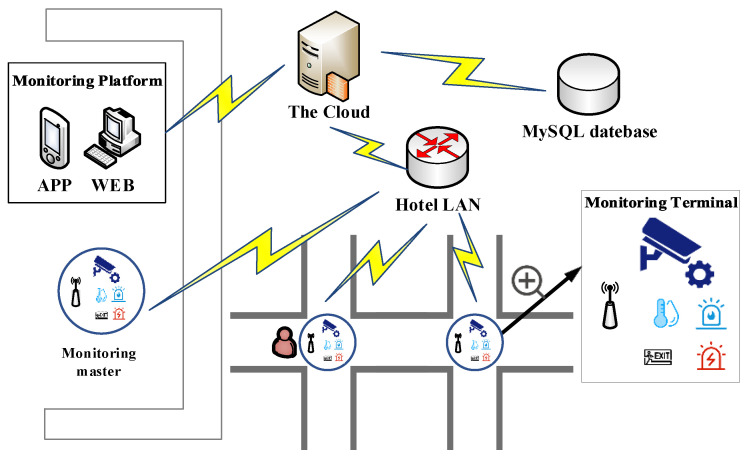
The overall architecture of the system.

The overall design architecture of the system is shown in Figure 1. The hotel face recognition system is divided into three parts, including monitoring device, the Cloud and management platform.

Monitoring devices are divided into a monitoring master at the Front Desk (FD) and multiple monitoring terminals are distributed in the entrance of the hotel corridors. Their designs are roughly the same. The monitoring device includes temperature and humidity detection, ultrasonic ranging and other sensor modules, as well as alarm buttons, dot matrix screens and so on. The function of FR is realized by the face recognition module (OpenMV). OpenMV, with STM32H743VIT6 as the core processor, is an open source machine vision project initiated by MicroPython, which aims to create a minimalist vision processing module.

The communication between system devices is roughly based on MQTT [22,23,24,25]. MQTT is often used to overcome the gap between different protocols. The monitoring master uses the rule engine of the Cloud to transmit the customer information to the user and management side, and stores information to the MySQL database. Only the monitoring master can upload the data to the Cloud with the Local Area Network (LAN). In other words, monitoring terminals only communicate with each other in the LAN. The management platform at the FD includes the APP and the WEB visual interface. Therefore, the manager can query customer information directly.

After entering the hotel, the customer needs to enter the identity information, such as facial photos, at the monitoring master at the FD to check in, and then the information is uploaded to the Cloud via the LAN using the Wireless Fidelity (WiFi) module. When the system provides intelligent guidance, the customer takes pictures in front of a terminal. The photos are compared with the image features stored in the Secure Digital (SD) card, and the results are displayed on the screen.

### 2.1. System Hardware Design

**Figure 2:**
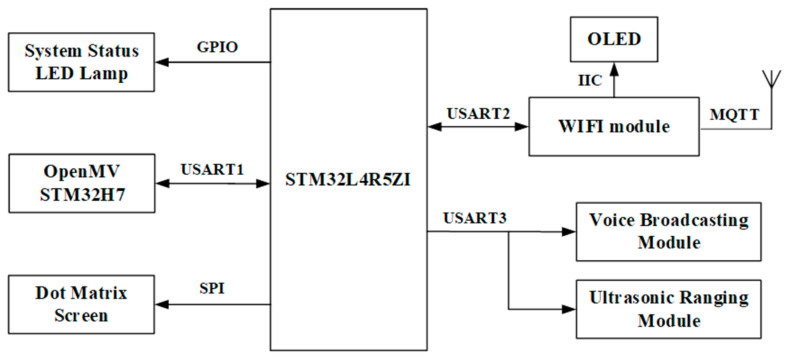
The overall frame diagram of the monitoring device.

The master and the terminal use the same hardware design. The main control part of the monitoring device uses STM32L4R5ZI as the core processor, which is responsible for communication. The monitoring device is equipped with ultrasonic ranging and other modules. The WiFi module uses ESP8266 of Lexin to communicate with temperature and humidity sensor to collect environmental parameters. The WiFi module shares information to terminals via the hotel LAN and uploads the log information to the Cloud. Managers can control hotel’s alarms through the WEB or the APP. The overall frame diagram of the monitoring device is shown in Figure 2.

**Figure 3:**
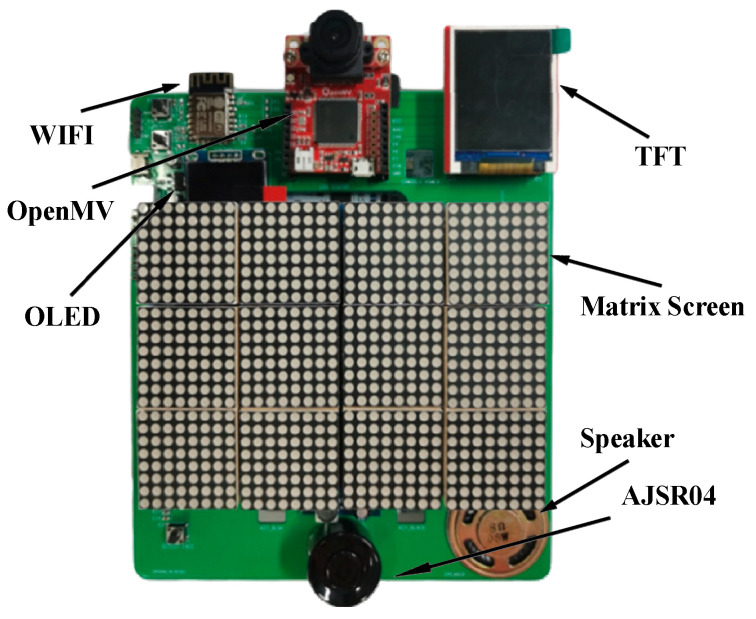
The finished product of the monitoring device.

The finished product of the monitoring device is shown in Figure 3. The STM32L4 chip is located on the backplane, and the OpenMV is installed directly above. The middle layer is equipped with 12 8 × 8 dot matrix screens to display guidance information, and the ultrasonic module is installed below. The upper left of the object is the WIFI module and the OLED screen, and the upper right is the TFT screen. The two screens are used for system testing and can be removed in actual use.

The system uses a 220 V adapter for power supply, and is connected to a 5 V DC power interface on the self-designed backplane. In total, 5 V supplies power to the voice broadcast module. In the hardware design, two sets of power supply voltage stabilizing and reducing modules are added. The voltage is regulated from 5 V to 3.3 V to power the two ARM processor cores. This power supply design ensures stable operation of the power drive system.

### 2.2. System Software Design

#### 2.2.1. Communication Protocol

The Cloud acts as the MQTT Broker, which is built by Aliyun ECS server. The Wi-Fi module and management platform of the monitoring master act as MQTT Client. They communicate with each other by subscribing/publishing topics.

At present, the most commonly used data format is JSON. JSON is a lightweight data exchange format. In network communication, JSON is used to transmit data efficiently. There are many monitoring terminals in the actual hotel management and the ID of the monitoring terminal is set to the corresponding MAC address. The ID is stored in a key value, such as “SiteID”, by which the master can determine the location of the terminal. The ID and other information are encapsulated in JSON for transmission.

#### 2.2.2. Monitoring Device

The monitoring master is responsible for the input of customer face information, sharing information with terminals and communication with the cloud server. The monitoring terminal is responsible for receiving customer information, face recognition and direction guidance. Monitoring devices and the hotel router form a LAN, which in turn form a star topology, in which a centralized communication strategy is implemented by the central node. In this system, the hotel router is used as the central node, and TCP/IP (transmission control protocol/Internet protocol) communication is used between devices and the hotel router to achieve high-efficiency “point-to-point communication”.

The code compilation of monitoring devices consists of two parts. One is the STM32 programming environment, mainly based on the C language of Keil MDK, and the other is OpenMV, equipped with a MicroPython interpreter to run Python. The wireless communication task of monitoring devices is based on the Wi-Fi module, which is equipped with TCP, MQTT and other protocol stacks.

Once turned on, a monitoring device (either master or terminal) completes initialization first, then it sends a ranging request to the ultrasonic ranging module every second. If there is a customer in front of the ultrasonic ranging module and the measured distance is between 0.4 m and 0.8 m, the monitoring device will take portraits of the customer. If the customer is at the FD, the customer can take photos before the monitoring master after the administrator confirming on the WEB. After the photos are processed by LBP, the master stores their feature vectors, and then shares them with other terminals through the hotel LAN. At the same time, the customer’s name, photos and other information are synchronizes to the Cloud. After the information is successfully shared, customer photos will be deleted.

Inside the SD card of the monitoring device, a room information table is established to store the room number (RoomNum), the corresponding location of the monitoring terminal (SiteID), the customer check-in status (Status) and the customer name (ClientIDx).

**Figure 4:**
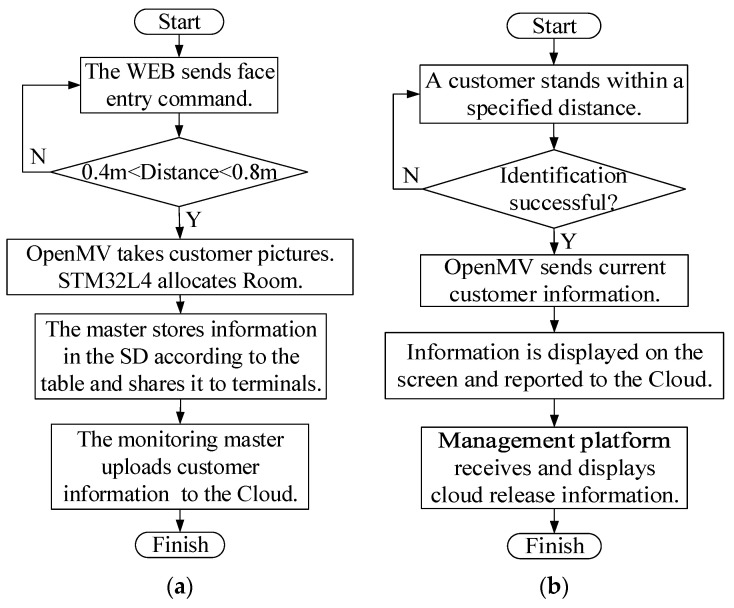
(**a**) Face information entry process. (**b**) Face information recognition process.

After the customer enters the facial information at the FD, OpenMV restarts automatically. STM32L4 automatically assigns a unique ClientID to the current customer, and assigns a unique RoomNum to distinguish and store according to the status of the room in the database. Because only the serial port interrupt can respond in the photo mode, the collision problem of multi-channel serial port interrupts can be avoided. The face information entry process is shown in Figure 4a.

The customer is identified in front of a monitoring terminal, and the customer’s location is known at this time, namely SiteID. STM32L4 compares the variance between the eigenvector of the photograph and the eigenvector of the original image. The STM32L4 of the monitoring terminal receives the ClientID through USART1 after successful recognition. The STM32L4 drives the dot matrix screen to guide customers according to the corresponding RoomNum table, and commands the voice broadcast module to work. After the identification, STM32L4 packages the current identification information into JSON format and uploads it to the Cloud when the monitoring master is idle. The flow chart of the system face information recognition is shown in Figure 4b.

In addition, when the environmental monitoring value is abnormal, the system automatically triggers the alarm. At the same time, managers conduct disaster alarm and turn on alarm lights through the APP or the WEB.

When the terminal is working, the ideal distance between the customer and the terminal is closely related to environmental factors such as lighting. When the lighting condition is ideal, the distance is about 0.4 m~0.8 m. At the same time, the voice broadcast module will remind the customer to adjust the front and rear position, which can reduce the influence of subjective factors.

The corridor light of the hotel is dim, and the resolution is not high, so illumination has a great influence on face recognition. The OpenMV recognition technology mainly adopts LBP algorithm which is robust to illumination and posture [26,27,28]. The LBP operator was proposed by Ojala et al. in 1996. For each pixel in the image, the size relationship between the central pixel and the surrounding pixel in the 3 × 3 neighborhood is compared, and the gray value of the pixel is converted into an octet binary sequence [29]. The LBP operator makes use of the relationship between the center point and the surrounding point to quantify it, which can more effectively eliminate the influence of light. The LBP operator can also be combined with Principal Component Analysis, Support Vector Machine and Deep Learning for recognition, which can further improve the recognition efficiency and effectiveness [30,31,32,33]. The path performance between network terminals and the central node is very important. The uploaded data is time-dependent, and a link congestion will affect the effectiveness and reliability of the whole system. This system adopts the matrix completion method [34,35] for low-cost network monitoring, which can recover the lost data with a small number of known samples, and can retransmit the data if the data loss is serious.

Ahonen T [29] supports that retaining the information about spatial relations is important. Thus, to retain the information, STM32H7 calculates the statistical histogram of LBP image, and then normalizes it to get one-dimensional feature vector. The monitoring master trains eigenvectors and encrypts them by matrix. The encrypted information is sent to monitoring terminals through the LAN.

The application scenario of this system belongs to the open-set face identification. The gallery consists of portrait photos of customers who have already checked in. Face images taken by terminals constitute the probe. Usually, the equal error rate (EER) is calculated when false accept rate (FAR) equals the false reject rate (FRR), and the performance of the system is relatively good at this time. Compared to closed-set tests, open-set tests add a set of impostors who are neither included in the probe or in the gallery.

#### 2.2.3. Management Platform

In order to achieve smooth management, MQTT Broker, MySQL Server and Node-RED platform are built on the server.

The APP is designed by Android Studio, and the APP interface not only displays environmental information, but also adds control buttons such as fire alarm, safety exit identification and so on. The monitoring master and terminals use ESP8266 to communicate with Android. After the customer checks in at the FD, the information is stored in the database synchronously, and the Cloud forwards the current customer’s status information to the APP. This system uses MySQL database to store customer information. The APP completes the creation and configuration of MySQL and MQTT clients through the official jar package. At the same time, the third-party package gson-2.8.6.jar is used to package and parse JSON format data.

Visual WEB interface is designed by Node-RED. Node-RED supports MQTT and MySQL, and carries many visual plug-ins such as vector graphics, charts, etc., which reduce the amount of programming. The network bar of the Node-RED node list contains “mqtt in” and “mqtt out” plug-ins, which can connect to the MQTT agent and subscribe to specified topics or publish messages. Node-RED is designed with message flow “msg.payload”, and the data structure is customer friendly. The IP address and port number of the bound MQTT Broker side are entered in the node properties. The structure will be encapsulated in the message flow, and the value corresponding to “client1” in the JSON data is called through the {{msg.payload.client1}}. For publishing messages, the data flow is encapsulated into JSON format and published by calling the Change node.

The MySQL client is labeled as “mysql” in the storage column of the node list. Similar to MQTT Broker, the IP address and port number on the MySQL server side are set. The “mysql” node contains the input and the output. The input uses a function to modify and query the database, and encapsulates the data in the data stream. After database addition, deletion, modification and query, the Node-RED obtains the output. The “text” plugin is placed in the node list to display the data on the dashboard of Node-RED.

## 3. System Testing and Analysis

**Figure 5:**
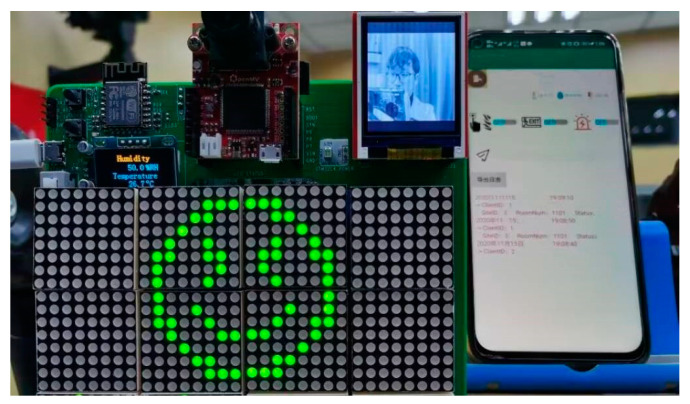
System test.

The power-up effect of the system is tested, as shown in Figure 5. After system initialization, the dot matrix screen displays “Welcome”. By default, the TFT screen displays the captured face in real time. The yellow LED flashes, indicating that the system is currently measuring distance. If a customer is within the target distance, the yellow LED stops flashing, the blue LED is lit, and the dot matrix screen prompts the status.

### 3.1. Data Entry

The system simulates the scene of customers checking in. At the FD of the hotel, OpenMV take portraits of the customer after the WEB interface sends face entry commands to the monitoring master. The dot matrix screen dynamically displays the current number of photos, and the voice module plays the current status synchronously. After the photos are taken, the system assigns a room to the customer in sequence and binds a unique ClientID.

### 3.2. Identification and Guidance Testing

**Figure 6:**
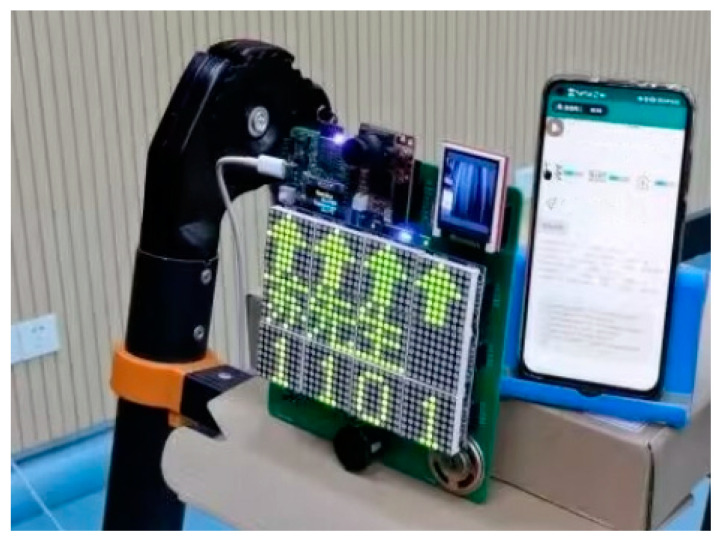
System performance. The dot matrix screen shows that “Mr. Su’s room number is 1101”, and the direction is forward. The APP displays check-in log, including customer name, RoomNum and other information.

To test the identification function of the monitoring terminal, after the customer is identified successfully, the dot matrix screen shows the customer’s room number and direction according to the previously assigned room (RoomNum) and location information. The voice module broadcasts at the same time and the APP receives status information. The recognition effect of this system is shown in Figure 6.

### 3.3. WEB Visual Iterface Testing

**Figure 7:**
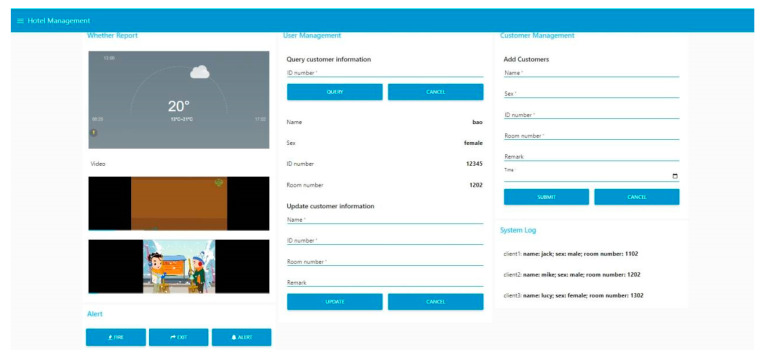
The Node-RED visual interface.

Information entered by hotel customers upon check-in is reported to the database, and it is displayed on the WEB. The functions of WEB include querying customer information, updating and adding information, etc. The system log displays the customer status in real time. The interface can also directly control the hotel emergency facilities such as alarms. The Node-RED visual interface is shown in Figure 7.

### 3.4. Algorithm Comparison

The information of 200 customers is entered into the system, and each customer takes ten photos as a sample. The 200 customers plus 10 unregistered were tested for identification. Photos taken are all processed to 224 × 224. Terminals compare the variance with all other samples to determine whether the image belongs to gallery. The experiment is based on open-set evaluation, and adopts FAR as a performance metric, as shown in Table 1. Next, run FR algorithms on the PC. Considering the computer processor will not be the latest version, the CPU of the PC chooses Pentium-G2120T and processor frequency is 2.7 GHz. The recognition accuracy of the system using the CNN is 96.2%. The LBP algorithm is similar to the PCA in terms of recognition accuracy. Next, run the algorithm on the development board. The system core STM32H743VIT6 is Cortex-M7, and its frequency is 480 Mhz. The running time of using LBP algorithm is much less than that of systems using CNN. Combined with the user experience, the LBP is suitable for the application environment of face recognition in hotel. There is a certain error in the recognition result using LBP, which is mainly due to the influence of lighting, background and other factors on OpenMV, but the error is within the range of acceptance. The eigenvalues of LBP recognition are about several thousand. If affected by the above factors, the range of eigenvalues will change in a large range. Eliminating the values with large differences in eigenvalues in each face recognition can improve the recognition rate of the system.

### 3.5. Security and Privacy

The expansion of IoT will also bring about many security and privacy issues. Low-end commercial products of IoT usually do not support strong security mechanisms. Various devices in the hotel are connected to the wireless sensor network; at this time, there is a greater risk of information leakage, and maybe in some cases they have become the target of malicious attacks. To reduce the risk of direct exposure of personal photos and protect customer privacy, photos are processed by LBP features in the monitoring master; the encrypted feature vectors are shared to each monitoring device. Next, the original images in the master are deleted. Information in terminals is only used for identification. The images taken by terminals after recognition will also be deleted. After customers checking out, information in monitoring devices will be deleted according to ID. The security of the Cloud is provided by a third-party platform. After system deployment, it is equipped with a physical shell to prevent physical intrusion. In the aspect of system software design, the program designs the memory card monitoring and alarm program. If SD is not detected, it indicates poor card contact or theft. Managers will receive a notification if the system detects an abnormal alarm. 

### 3.6. The Complexity of Time and Memory

Combined with the advantage of edge computing, face recognition modules are deployed at the edge of the system network, which greatly improves the processing efficiency and reduces the load on the central server, so as to reduce running time and memory footprint. In order to improve the operation efficiency, the system uses the dual ARM core architecture. The image training time and network transmission time do not affect the system guidance. The monitoring terminals are responsible for picture recognition, and transmit processed and encrypted picture features. The characteristic of a matrix is about 10 kb, and the SD card with 4 G memory is selected. It can store thousands of customer information, which can meet the needs of the hotel.

## 4. Conclusions and Future Work

With the progress of IoT technology and big data analysis technology, urban construction is more intelligent. In order to improve the operational efficiency of the hotel industry and optimize the customer experience, the system puts forward a set of low-cost wisdom upgrade schemes based on the investigation of the current situation of hotel management. The system adapts the dual ARM core architecture. STM32H7 runs the FR algorithm independently, and STM32L4 acts as the master controller for data communication with other peripheral devices. Monitoring devices automatically enter sleep mode in idle time to prevent false detection, which significantly reduces power consumption. The system uses the MQTT protocol to realize the information exchange between monitoring devices and the management platform, achieving real-time storage and query of hotel customer information. This system can be adapted to different kinds of hotel environments, according to the layout. The competitiveness of the hotel can be enhanced by controlling operating costs, which can enhance the sustainability of hotel development.

However, there are still many areas that need further research:

- Combined with the wake-up times of devices, the hotel can set more vending machines in the places with large passenger flow to increase the additional income of the hotel.
- The diffuse reflection light and other biometric technology can be used for face recognition, which can accurately obtain facial characteristics to carry out a real person static check. This technology has strong anti-interference and can effectively avoid malicious behavior using photo recognition.
- If the number of customers increases, three-dimensional face recognition neural network algorithms can be used to process pictures, which can further improve the efficiency of the system.

## Figures and Tables

**Table 1:** Recognition Rate and running time (Open-set evaluation, FAR = 1%).

Method	Running Time (on PC)	TAR@FAR (on PC)	Running Time (on Board)	TAR@FAR (on Board)
PCA	0.74 s	94.6%	3.7 s	93.2%
CNN	1.24 s	96.2%	>15 s	95.0%
LBP	0.63 s	93.8%	1.2 s	93.0%

## Data Availability

Data are contained within the article.
